# Oxygen Metabolic Responses of Three Species of Large Benthic Foraminifers with Algal Symbionts to Temperature Stress

**DOI:** 10.1371/journal.pone.0090304

**Published:** 2014-03-03

**Authors:** Kazuhiko Fujita, Takaaki Okai, Takashi Hosono

**Affiliations:** 1 Department of Physics and Earth Sciences, University of the Ryukyus, Okinawa, Japan; 2 Department of Earth and Planetary Science, Graduate School of Science, The University of Tokyo, Tokyo, Japan; University of Connecticut, United States of America

## Abstract

Water temperature affects the physiology of large benthic foraminifers (LBFs) with algal symbionts dwelling in coral reef environments. However, the detailed physiological responses of LBF holobionts to temperature ranges occurring in their habitats are not known. We report net oxygen (O_2_) production and respiration rates of three LBF holobionts (*Baculogypsina sphaerulata* and *Calcarina gaudichaudii* hosting diatom symbionts, and *Amphisorus kudakajimensis* hosting dinoflagellate symbionts) measured in the laboratory at water temperatures ranging from 5°C to 45°C in 2.5°C or 5°C intervals and with light saturation levels of ∼500 µmol m^−2^ s^−1^. In addition, the recovery of net O_2_ production and respiration rates after exposure to temperature stress was assessed. The net O_2_ production and respiration rates of the three LBF holobionts peaked at ∼30°C, indicating their optimal temperature for a short exposure period. At extreme high temperatures (≥40°C), the net O_2_ production rates of all three LBF holobionts declined to less than zero and the respiration rates slightly decreased, indicating that photosynthesis of algal symbionts was inactivated. At extreme low temperatures (≤10°C for two calcarinid species and ≤5°C for *A. kudakajimensis*), the net O_2_ production and respiration rates were near zero, indicating a weakening of holobiont activity. After exposure to extreme high or low temperature, the net O_2_ production rates did not recover until the following day, whereas the respiration rates recovered rapidly, suggesting that a longer time (days) is required for recovery from damage to the photosystem by temperature stress compared to the respiration system. These results indicate that the oxygen metabolism of LBF holobionts can generally cope well with conditions that fluctuate diurnally and seasonally in their habitats. However, temporal heat and cold stresses with high light levels may induce severe damage to algal symbionts and also damage to host foraminifers.

## Introduction

Climate change, in particular global warming and ocean acidification, adversely affect coral reefs and reef calcifying organisms [1], [2]. The projected increases in atmospheric carbon dioxide and temperature for this century exceed the conditions under which coral reefs have flourished over the past half-million years [2], [3]. Rising water temperatures caused by global warming induce bleaching (defined as the loss of photosynthetic microalgae and/or their photopigments) of corals and other reef organisms with symbiotic algae [4]. Mass bleaching events have occurred in association with episodic elevated water temperatures in recent decades [5], and bleaching events are likely to become a chronic phenomenon in many reef areas in the coming decades [6]. Bleaching at elevated temperature is related to the photoinhibition of algal symbionts [7], [8]. Temperature limits the rate of electron transport in the photosystems and the turnover of associated D1 proteins [9]. As a result, the rate of excitation (light capture by reaction centers) exceeds the rate of light utilization (photochemistry). This excess energy results in a buildup of reactive oxygen species (ROS) [10], [11]. The overproduction of ROS causes damage to the photosynthetic apparatus of symbionts as well as to host cells [12], [13].

Large benthic foraminifers (LBFs) are unicellular calcifying reef organisms that can form symbiotic relationships with a range of different microalgae [14]. An LBF-microalgal holobiont acts as a primary and carbonate producer [15]. Temperature is one of the primary environmental factors that affects the physiology and ecology of LBF holobionts [16]. Growth rates of host LBFs are negatively affected by prolonged periods of high water temperature [17]–[19]. The geographical distribution, and particularly the latitudinal limits of LBFs, is related to the lowest water temperature in winter [20], [21]. The chemical composition of LBF shells (*e.g.*, oxygen isotopes, Mg/Ca ratio) is correlated with temperature [22], [23]. LBFs with algal symbionts exhibit bleaching caused by high temperature [17], [18], [24] and/or UV radiation [25]–[27].

Some LBFs such as dinoflagellate-bearing *Marginopora*, and diatom-bearing *Amphistegina*, *Calcarina*, and *Heterostegina* can be negatively affected by exposure to temperatures above a threshold value, which is often a few degrees higher than the local summer maxima [17], [18], [28]. At elevated temperatures (>30°C), algal symbionts, irrespective of symbiont type, exhibited decreased concentrations of chlorophylls and other photopigments [17], [18], [24], [29], a decline in the maximum quantum yields of Photosystem II (PSII; *Fv*/*Fm*) and other photophysiological parameters [17], [18], [24], [29], reduced numbers of viable symbionts and high numbers of deteriorating symbionts [17], [26], and decreased levels of RuBisCO protein (the enzyme responsible for fixing CO_2_) [30]. Host foraminifers became inactive [24], grew slowly [17]–[19] and tended to have increased mortality [18]. LBF holobionts exhibited bleaching [17], [26], decreased net O_2_ production rates and increased respiration rates [17], [18], decreased organic carbon (C) and nitrogen (N) content, and molar C/N ratios [18]. In contrast, the physiological responses of LBF holobionts to low temperatures have not been fully determined. Zmiri et al. [31] investigated the phototaxis and thermotaxis of diatom symbiont-bearing *Amphistegina* species at various temperatures in the laboratory, and observed that two species of *Amphistegina* (*A. radiata* and *A. madagascariensis*) did not move at temperatures ≤12°C and ≤16°C, respectively.

In LBF holobionts, oxygen (O_2_) is produced under light conditions by the photosynthesis (PSII) of algal symbionts, while O_2_ is consumed in dark conditions due to the respiration of the host and symbionts [15]. Photosynthesis (O_2_ production) and respiration (O_2_ consumption) rates tend to be affected by temperature because photosynthetic carbon assimilation and the electron transport of photosynthesis and respiration are enzymatically controlled, which is a temperature-dependent process [32]. Thus, the maximum photosynthetic rate at light saturation increases with temperature [33]. Under conditions of light saturation, photosynthetic responses to temperature are not linear over a wide range, and a temperature optimum exists, below and above which the photosynthetic rate decreases [32], [33]. Respiration generally increases with temperature [33]. Thus, the response of photosynthesis and respiration to temperature expressed as O_2_ production and consumption rates reflect the response of LBF holobiont activity to temperature stress. An understanding of their optimum temperature and tolerances would improve our knowledge on the ecology of LBFs, including their distribution, seasonality, and life history. Therefore, we undertook laboratory experiments to measure changes in the O_2_ metabolic rate of LBF holobionts at different temperature ranges in their habitats.

## Materials and Methods

### Target species, collection, and sampling sites

Three species of LBFs with algal symbionts were selected for this study: *Baculogypsina sphaerulata* (Parker and Jones 1840), *Calcarina gaudichaudii* d′Orbigny 1840, both of which host diatom symbionts, and *Amphisorus kudakajimensis* (Gudmundsson 1994), which hosts dinoflagellate symbionts. These species were chosen because their photosynthesis–irradiance (P–I) curves have previously been determined [15], and they are abundant in the intertidal to upper subtidal zones of coral-reef flats in the Northwest Pacific [34] (for the detailed taxonomy, test morphology, biology and ecology, refer to Hohenegger [35]).

Foraminifers used for this study were collected from reef flats around the Okinawa Islands (Japan) from August to December 2006. For *B. sphaerulata* and *C. gaudichaudii*, living individuals were collected from algal turfs in a tide pool (mean water depth: ∼0.5 m) on the east coast of Ikei Island (26.40N, 128.00E). For *A. kudakajimensis*, living individuals were collected in a nearshore macroalgal zone (mean water depth: ∼1 m) at the Hizushi reef flat, southwest of Akajima Island (26.19N, 127.27E) or at the Zampa reef flat, west of Okinawa Island (26.43N, 127.71E), depending on the season. Since all the three sampling sites described above are open to the public, no specific permissions are required for these locations/activities except for catching fishery resources. The studied species are not designated as endangered or protected species at the present time. However, we attempted to keep the number collected to a minimum.

In the laboratory, living individuals were isolated under the microscope, cleaned with a fine brush, and rinsed several times with filtered (0.22-µm) seawater (FSW). These living individuals were maintained in a petri dish filled with FSW and placed on a wave shaker (Mini-shaker, LMS, Tokyo, Japan) in an incubator until measurements were made. The incubator was kept at 25°C under a 12-h light/dark cycle with a light intensity (photosynthetic photon flux density) of ∼100 µmol m^−2^ s^−1^, which was less than the minimum saturation level of the studied species (180–240 µmol m^−2^ s^−1^) [15].

As Okinawan coral reefs are located in the subtropical oceanic climate zone influenced by the Asian monsoon, the water temperature fluctuates seasonally (Figure S1). In shallow subtidal reef flats, the mean annual water temperature is ∼25°C, with a maximum of 31°C in summer and a minimum of 17°C in winter. The diurnal temperature fluctuation is small (<2°C) throughout the year. In shallow tidal pools, water temperature varies more substantially than at the subtidal zone. The mean annual water temperature (∼26°C) is similar to that in shallow subtidal reef flats, but the maximum water temperature at midday exceeds 40°C at low tide in summer, and the minimum water temperature at midnight decreases to 10°C at low tide in winter. The diurnal fluctuations in water temperature are substantial (5–13°C) throughout the year.

### Measurement protocol

We measured the changes in dissolved oxygen (DO) levels before and after a closed incubation of LBF individuals at water temperatures ranging from 5°C to 45°C in 2.5°C or 5°C intervals. To measure the population-level metabolism, ∼300 individuals of *B. sphaerulata* or *C. gaudichaudii*, and 10–25 individuals of *A. kudakajimensis* were pooled for measurements. The same foraminiferal individuals were used for several measurements at different temperatures (referred to as a trial). Measurements were made at one temperature per day and a recovery phase was provided that lasted until the following day. The temperature range for each trial was either 5–25°C, 15–35°C, or 25–45°C, which represents the lower-limit, normal and upper-limit temperature ranges, respectively, observed in the LBF habitats. Measurements started at 25°C (near the mean annual temperature at the sampling sites), and then the temperature was adjusted up or down in 2.5°C or 5°C intervals each day. The total numbers of trials were eight for *B. sphaerulata* and *C. gaudichaudii*, and nine for *A. kudakajimensis*. The total number of measurements per temperature (replicates) was a maximum of nine, depending on temperature. Furthermore, to assess the recovery of LBF holobionts after exposure to temperature stress, net O_2_ production and respiration rates at 25°C were measured on the following day, and compared with the initial rates measured at 25°C at the start of each trial. All measurements for each trial were completed within 9 days of collection.

Water temperature was controlled inside a stainless steel water bath using the combination of a thermostat (TR-2A, As One, Tokyo, Japan) and a cooling unit (100TCN, Iuchi, Tokyo, Japan), with a precision of ±0.2°C. Other environmental variables were maintained constant during the measurements. Light intensity was set to greater than the light saturation level of the studied species (500±50 µmol m^–2^ s^–1^), which represents the average annual light intensity during daytime in the study area. This was because the aim of this study was to clarify the temperature responses of LBF holobionts in their habitats. The light was provided from above using a neutral density-filtered halogen lamp (PCS-NHF150, NPI, Tokyo, Japan).

A closed incubation system was used to measure changes in DO levels. Prior to measurements, a 2-L bottle of FSW was immersed in the water bath and stirred until the temperature inside the bottle was identical to that in the water bath. The DO in FSW was recorded as DO before incubation (DO_before_). DO was measured using a Clark-type dissolved oxygen electrode (model 810Aplus, Thermo Electron, Waltham, MA, USA). Then live individuals of each LBF species (∼300 individuals of *B. sphaerulata* or *C. gaudichaudii*, and 10–25 individuals of *A. kudakajimensis*) were transferred into a beaker containing FSW and kept in a water bath for 30 min to acclimatize before incubation. These individuals were then placed onto a nylon mesh (100-µm openings) in a clear, airtight container (culture cup) with a clear lid (composed of polyethylene terephthalate, 8.5 cm in diameter and 4 cm in height) that contained 160 mL of FSW and was sealed with paraffin. Three sets of culture cups with foraminifers (*i.e.*, one culture cup for each species) as well as a blank cup with no foraminifers were prepared for each trial. The culture cups were placed on a waterproof magnetic stirrer in a water bath (MICRO+Telemodul 40C, H+P Labortechnik AG, Oberschleissheim, Germany), and seawater in the culture cup was continuously stirred by a small magnetic bar (30 mm in length) placed under the mesh (300 rpm).

Measurements in the light and dark at each temperature were made on the same day. Measurements were first taken during dark incubation for 2 h. After the dark incubation, DO levels in the incubated seawater were measured and recorded as DO after incubation in the dark (DO_after_dark_). Individuals were then acclimatized under light conditions for 30 min. Seawater in a cultured cup was replaced with fresh FSW and incubated under light conditions for 2 h. After the light incubation, DO in the incubated seawater was recorded as DO_after_light_. After measurements in the light, foraminiferal individuals were maintained in the incubator under the conditions described above until the following day to provide foraminifers with a recovery phase before being measured at the next temperature.

The rate of net O_2_ production or respiration was calculated as:

O_2_ (mg L^–1^)  =  DO_after_ – DO_before_ – ΔDO_blank_,

where DO_after_ and DO_before_ are the DO after and before incubation under either light or dark conditions, respectively, and ΔDO_blank_ is the change in DO in a blank cup before and after incubation. ΔDO_blank_ was generally one or two orders of magnitude smaller than the change in DO in culture cups with foraminifers. If ΔDO_blank_ was larger than ±0.1 mg L^–1^, due to either a leakage from the imperfect seal of the culture cup or O_2_ consumption by bacteria or other contaminants, DO measurements on the same day were omitted from the data.

To assess the recovery of a LBF holobiont after exposure to temperature stress, DO changes at 25°C under light and dark conditions were measured on the day following incubation at extreme low or high temperatures (≤15°C or ≥35°C). Then those rates (referred to as *P*_25:after_, *R*_25:after_) were compared with those initially measured at 25°C at the start of each trial (*P*_25:initial_, *R*_25:initial_).

After all measurements were completed, foraminiferal individuals were rinsed with distilled water and air-dried. The dry weight of individuals, which consists of shells and protoplasm, was measured using a microbalance with a precision of ±0.1 mg (BP121S, Sartorius AG, Göttingen, Germany). Net O_2_ production and respiration rates were converted into mole units per dry weight per hour.

### Data analysis

Generally, enzyme-catalyzed reactions such as photosynthesis and respiration are temperature-dependent and nonlinear [32]. To quantify the response of photosynthesis/respiration of LBF holobionts to temperature, a generalized additive model (GAM) with temperature as an explanatory variable was used. The linearity/nonlinearity of the response was examined by the likelihood ratio test using a simple linear model with temperature as an explanatory variable.

The recovery of LBF holobionts after exposure to temperature stress was assessed by *P*_25:after_ – *P*_25:initial_ and *R*_25:after_ – *R*_25:initial._ Because these differences displayed a nonlinear response (see Results), the effect of exposure temperature on the recovery was tested with the following linear model:

*P*_25:after_ – *P*_25:initial_ or *R*_25:after_ – *R*_25:initial_ ∼*T* ^2^ + *T* + *e*,

where *T* is the exposure temperature and *e* is an error term. When the exposure temperature has no effect on the recovery process of LBF holobionts, *P*_25:after_ – *P*_25:initial_ or *R*_25:after_ – *R*_25:initial_ will be distributed around zero (a nonsignificant regression line or a line with a slope close to zero). However, if the exposure temperature affects the recovery, the response of *P*_25:after_ – *P*_25:initial_ or *R*_25:after_ – *R*_25:initial_ can be detected as a significant monotonic increase/decrease line (i.e. significant first-order term) or a quadratic curve (i.e., significant second-order term) against the exposure temperature.

All statistical analyses were performed using R 2.14 software [36] and the MGCV package [37].

## Results

### Response to temperature

The likelihood ratio test revealed a significant nonlinear relationship between net O_2_ production/respiration rates and temperature under both light and dark conditions (Table 1).

**Table 1:** Summary of the analysis of temperature effects on net photosynthesis and respiration for each of three species of large benthic foraminifers with algal symbionts using a linear model (LM) and a generalized additive model (GAM) with only temperature as an explanatory variable, and the results of a likelihood ratio test (LRT) for the two models.

		LM					GAM						LRT		
Species	Response variable	Estimate	*t*	*p*	Resid df	Resid dev	edf	Ref.df	*F*	*p*	Resid df	Resid dev	*F*	*p*	
*Baculogypsina sphaerulata*	Net photosynthesis	0.12	1.81	0.080	35.00	491.17	5.97	7.09	23.67	**<0.001**	30.03	79.38	31.347	**<0.001**	GAM>LM
	Respiration	-0.05	-2.49	**0.018**	35.00	46.66	3.14	3.96	5.31	**0.002**	32.86	31.84	7.1362	**0.002**	GAM>LM
*Calcarina gaudichaudii*	Net photosynthesis	0.05	1.22	0.233	35.00	170.38	6.76	7.77	24.02	**<0.001**	29.24	23.60	31.554	**<0.001**	GAM>LM
	Respiration	-0.03	-3.70	**<0.001**	35.00	8.79	2.89	3.64	7.45	**0.000**	33.12	6.42	6.4645	**0.005**	GAM>LM
*Amphisorus kudakajimensis*	Net photosynthesis	0.01	0.11	0.913	42.00	1954.05	5.36	6.49	13.99	**<0.001**	37.64	554.22	21.816	**<0.001**	GAM>LM
	Respiration	-0.12	-3.50	**0.001**	42.00	191.72	2.62	3.32	5.85	**0.002**	40.39	162.87	4.4301	**0.025**	GAM>LM

Resid df: residual degree of freedom, Resid dev: residual deviance, edf: estimated degree of freedom, Ref. df: reference degree of freedom, *F*: F value, *p*: p value.

Significant p values are indicated in bold.

**Figure 1:**
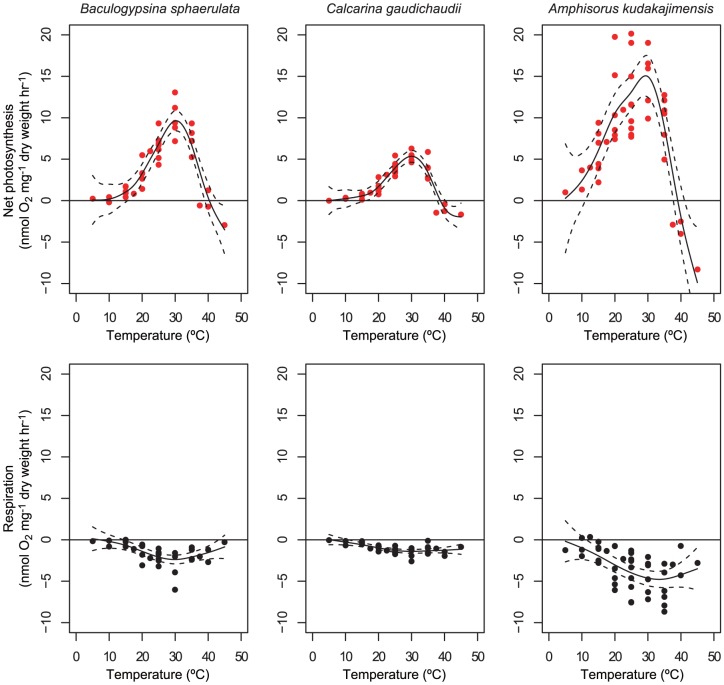
Net photosynthesis and respiration rates of large benthic foraminifers with algal symbionts at different water temperatures. Filled circles indicate observed data. The solid line is the curve fitted by a generalized additive model (GAM) with temperature as an explanatory variable. Dashed lines are 95% confidence intervals.

Under light conditions, all three LBF holobionts displayed a maximum net O_2_ production rate at ∼30°C (Figure 1, Table 2). At temperatures higher than 30°C, net O_2_ production rates of all three LBF holobionts declined and were less than zero at ≥40°C. At temperatures lower than 30°C, the net O_2_ production rates of all three holobionts also declined, but never became less than zero. The net O_2_ production occurred at ≤10°C for *A. kudakajimensis*, whereas the net O_2_ production was close to zero at ≤10°C for the two calcarinid species (*B. sphaerulata* and *C. gaudichaudii*).

**Table 2:** Summary of the peak point of net O_2_ production and respiration rates with 95% confidence interval (CI) and their temperatures inferred from the generalized additive model.

		Temperature	Peak rate	Lower limit CI	Upper limit CI
Species		(°C)	(nmol O_2_ mg^-1^ dry wt. hr^-1^)		
*Baculogypsina sphaerulata*	Net photosynthesis	30.3	9.64	8.42	10.86
	Respiration	29.8	-2.38	-2.91	-1.85
*Calcarina gaudichaudii*	Net photosynthesis	30	5.35	4.63	6.07
	Respiration	30	-1.39	-1.62	-1.17
*Amphisorus kudakajimensis*	Net photosynthesis	29.3	15.06	12.62	17.50
	Respiration	33.4	-4.78	-5.75	-3.80

Under dark conditions, the respiration rate of three LBF holobionts reached a maximum at ∼30°C (*B. sphaerulata* and *C. gaudichaudii*) or 33°C (*A. kudakajimensis*), from which they tended to decrease at higher and lower temperatures (Figure 1, Table 2). The respiration rate dropped to near-zero at ≤15°C for *B. sphaerulata* and *C. gaudichaudii* and at ≤10°C for *A. kudakajimensis*. The respiration rate at high temperatures also tended to decline, but did not decrease greatly compared with the decrease at lower temperatures.

### Recovery

**Figure 2:**
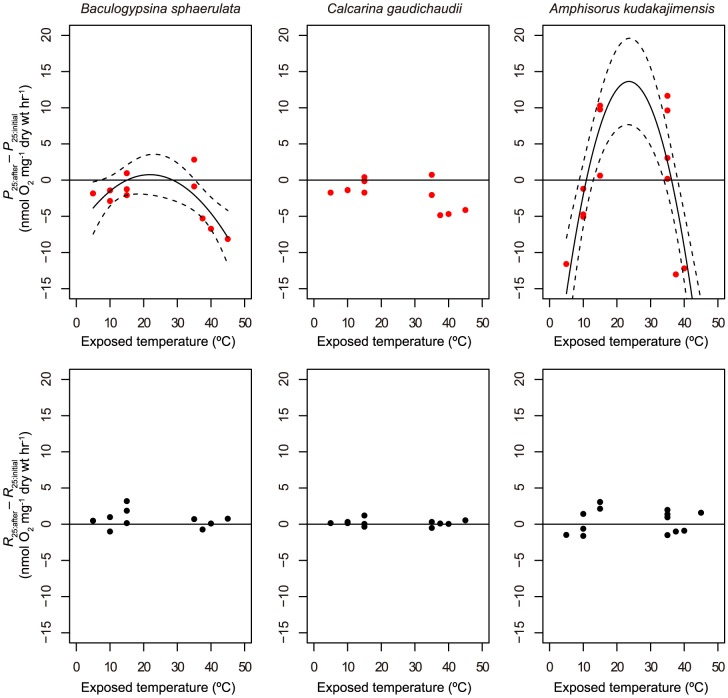
Metabolic recovery of large benthic foraminifers with algal symbionts after exposure to temperature stress. Net O_2_ production and respiration rates at 25°C on the day following exposure to temperatures stress (≤15°C or ≥35°C) (*P*_25:after_, *R*_25:after_) were compared with those initially measured at 25°C at the start of each trial (*P*_25:initial_, *R*_25:initial_). Filled circles indicate observed data. The solid line is the significant regression curve estimated by the linear model with temperature as an explanatory variable. Dashed lines are the 95% confidence intervals of the curve.

After exposure to extremely high (≥40°C) or low (≤10°C) temperatures, the differences in *P*_25:after_ – *P*_25:initial_ were less than zero for two holobionts (*B. sphaerulata* and *A. kudakajimensis*) (Figure 2). For *A. kudakajimensis*, the differences in *P*_25:after_ – *P*_25:initial_ after 35°C and 15°C were above zero. Thus, a significant second-order relationship with exposure temperature was observed for these two holobionts (Table 3). A significant pattern did not occur for *C. gaudichaudii*, but as for *B. sphaerulata*, a tendency for a decline after exposure to extreme temperatures was detected. The differences in *R*_25:after_ – *R*_25:initial_ were small, and statistical analyses also indicated no significant responses to *T* and *T* ^2^ for all three holobionts.

**Table 3:** Summary of linear models of the metabolic recovery of large benthic foraminifers with algal symbionts at different temperatures (*T*).

Species		Variables	SS	*df*	*F*	*p*
*Baculogypsina sphaerulata*	Net photosynthesis	T^2^	35.0	1	5.99	**0.040**
		T	26.1	1	4.47	0.067
		Residuals	46.7	8		
	Respiration	T2	0.9	1	0.57	0.471
		T	0.7	1	0.43	0.529
		Residuals	12.0	8		
*Calcarina gaudichaudii*	Net photosynthesis	T2	7.4	1	3.31	0.106
		T	4.6	1	2.06	0.189
		Residuals	17.9	8		
	Respiration	T2	0.1	1	0.22	0.654
		T	0.1	1	0.25	0.629
		Residuals	1.9	8		
*Amphisorus kudakajimensis*	Net photosynthesis	T2	1066.5	1	33.85	**0.000**
		T	962.6	1	30.55	**0.000**
		Residuals	346.6	11		
	Respiration	T2	5.9	1	1.98	0.187
		T	5.7	1	1.92	0.194
		Residuals	32.7	11		

SS: sum of square, df: degree of freedom, *F*: F value, *p*: p value.

Significant p values are indicated in bold.

## Discussion

This study revealed the effect of fluctuating water temperatures, such as those that occur in the field, on net O_2_ production and respiration in LBF holobionts. Their recovery following short-term exposures to temperature stress was assessed. 30°C was found to be the optimal temperature for net photosynthesis and respiration of the studied LBF holobionts. This result is consistent with a previous culture experiment on dinoflagellate-bearing *Marginopora vertebralis*, which showed that net O_2_ production rates remained high between 28°C and 32°C and declined at 34°C under the same seawater pH and high light levels (300 µmol photons m^–2^ s^–1^) [17]. The maximum quantum yield of PSII (*Fv*/*Fm*), photopigments (Chl a, Chl c2), symbiont density, and host growth (calcification rate) also exhibited similar trends, indicating less damage below 30°C but severe damage of the holobiont at 34°C. Other culturing studies of the same and similar taxa also demonstrated that photophysiological parameters and photopigments of algal symbionts, and the growth of LBF hosts were not affected below a temperature of 30°C and were negatively affected within several days when temperatures were over 30°C [18], [24], [26], [29].

At extreme high temperatures (≥40°C), the net O_2_ production rates of all three LBF holobionts declined to less than zero, and the respiration rates decreased slightly to become comparable to the former rates, indicating that photosynthesis in the algal symbionts was inactivated at ≥40°C. In addition, after exposure to extreme high temperatures (≥40°C), net O_2_ production rates did not recover until the following day, whereas the respiration rates recovered rapidly, suggesting that a relatively long time period (days) is required for recovery from damage to the photosystem by heat stress, compared to the respiration system. No comparable studies have been conducted to investigate the effect of temperatures over 40°C and the subsequent recovery of the photosynthesis and respiration of LBF holobionts. However, a relatively long recovery time for photosynthesis would be consistent with the observed 1–2 day recovery of PSII (*Fv*/*Fm*) of algal symbionts (diatoms, dinoflagellates, and rhodophytes) in seven LBF species after exposure to the PSII herbicide diuron [38]. A short pulse of extreme high temperature at high light levels may cause overexcitation and the subsequent production of ROS, which causes proteins/enzymes to denature and the photosynthetic apparatus to be destroyed [30], as demonstrated in reef corals [7]-[9], [11]-[13]. However, the relatively quick recovery of the respiration system suggests that the damage to host foraminifers is less severe and that the host may have some resilience against short pulses of temperature stress.

At extreme low temperatures (≤10°C for two calcarinid species and ≤5°C for *A. kudakajimensis*), net O_2_ production and the respiration rates of all three LBF holobionts was close to zero, indicating a weakening of holobiont activity. In addition, after exposure to extreme low temperatures, the net O_2_ production rates did not recover until the following day, whereas the respiration rates recovered rapidly, suggesting that a relatively long time period (days) is also required for recovery from damage to the photosystem by cold stress, compared to the recovery of the respiration system. The implications of this study are similar to those of thermotaxic experiments [31]. Diatom symbiont-bearing hyaline species (*Amphistegina radiata* and *Amphistegina madagascariensis*) did not move at temperatures ≤12°C and ≤16°C, respectively. When returned to moderate temperatures, individuals moved again except for those exposed to a temperature of 4°C. The falloff of net O_2_ production and respiration with decreasing temperature may be attributable to the enzyme reactions associated with the carbon assimilation and respiration systems working progressively more slowly as temperatures decrease [32], [33]. Consequently, photodamage may have occurred by severe cold stress, as reported in reef corals with dinoflagellate symbionts [39].

We also identified the physiological differences between *A. kudakajimensis* (porcelaneous foraminifers with dinoflagellate symbionts) and two calcarinid species (hyaline foraminifers with diatom symbionts). Dinoflagellate-bearing *A. kudakajimensis* had a higher maximum net O_2_ production and respiration rate, and a greater tolerance to low temperatures than diatom-bearing calcarinids. Previous studies have also demonstrated that net O_2_ production rates at light saturation levels were higher in porcelaneous foraminifers with dinoflagellate symbionts than in hyaline foraminifers with diatom symbionts [15], [40]. Photosynthesis measurements using the maximum quantum yields of PSII (*Fv*/*Fm*) demonstrated that diatom-bearing taxa were more sensitive to high temperatures than dinoflagellate-bearing taxa [29]. Photopigment composition also differs between symbiont types; dinoflagellates contain higher concentrations of photoprotective pigments (diadinoxanthin) [38]. The xanthophyll cycle, which is a photoprotective anti-oxidative mechanism, may allow the dinoflagellate-bearing species to live under high light conditions without suffering oxidative damage [41]. A higher net O_2_ production rate of dinoflagellate-bearing *A. kudakajimensis* compared with diatom-bearing species likely reflects a higher photodamage threshold of the former species than the latter species under light and temperature stresses. Furthermore, shell structural differences possibly affect the response time to temperature changes; *A. kudakajimensis* is a large species with a thick, nonporous shell, while the two calcarinid species are small species with porous shells.

The LBFs examined in this study mainly live attached to macroalgae in the intertidal zone (the two calcarinid species) and shallow subtidal zone (*A. kudakajimensis*) of coral reef flats in the Northwest Pacific [34]. These environments generally attain their highest temperature and light intensity conditions during low tides at daytime in summer, and their lowest temperatures during low tides at nighttime in winter. The temperature tolerance and recovery of the LBF holobionts revealed in this study indicate that the oxygen metabolism of LBFs can generally cope well with conditions that fluctuate diurnally and seasonally by adjusting their photosynthetic efficiency [41], [42] and their positioning on the substratum [41], [43]. However, in periods of temporary temperature stress, when temperatures can exceed the threshold for oxygen metabolism, severe heat and cold stresses with high light levels may induce the inactivation of photosystems, bleaching [17], [26], and reduce energy storage [18]. These damages to algal symbionts would lead to a lack of movement [24], [31], reduced growth [17], [18], changes in symbiont genotypes [44], increased susceptibility to bacterial/algal infection, disease [25], [26], and finally the death of host foraminifers [18].

The results of this study are also consistent with the seasonality [45]-[47] and geographical distribution [16], [20], [21] of the LBF species studied. The life span of the two calcarinid species and *A. kudakajimensis* are estimated to be ∼16–18 months, and 12 months, respectively. A large number of juveniles are reproduced asexually during late spring and early summer, increasing in shell size while their population density decreases gradually [45]-[47]. The timing of asexual reproduction may be related to increased water temperatures during spring and summer, and their growth rates and longevity may be to seasonal temperature changes. The latitudinal limits of *B. sphaerulata* and *Calcarina* spp. are correlated with the lowest winter sea surface temperatures of ∼21°C and 23°C, respectively, while the latitudinal limit of *Amphisorus hemprichii* is related to the lowest winter sea surface temperature of 17°C [20], [21]. Our results revealed the short-term physiological responses of LBF holobionts to water temperature. Future studies on the physiological effects of stressors in combination with longer-term culture experiments will improve our understanding of the responses of LBF holobionts in a changing environment.

## Supporting Information

Figure S1 **Water temperature at sampling sites.** A, a shallow subtidal reef flat at Akajima Island from 1 May 2006 to 30 April 2007. B, an intertidal tidal pool at Ikei Island from 22 May 2012 to 22 April 2013. Water temperature was recorded using a submersible data logger (HOBO Pendant Temperature/Light Data Logger with precision of ± 0.5°C).
(EPS)
